# Virtual implantation of Derivo Embolization Devices using PreSize Neurovascular software: Accuracy evaluation and size selection comparison study

**DOI:** 10.1177/15910199231202272

**Published:** 2023-10-08

**Authors:** Ngoc T. Ngo, Fabian Flottmann, Francesco Iori, Mirko Bonfanti, Aikaterini Mandaltsi, Jens Fiehler, Maxim Bester

**Affiliations:** 1 Department of Diagnostic and Interventional Neuroradiology, University Medical Center Hamburg-Eppendorf, Hamburg, Germany; 2 Oxford Heartbeat Ltd, London, UK

**Keywords:** Device selection, simulation software, intervention planning, aneurysm treatment, flow diverter sizing

## Abstract

**Background:**

Evaluating the deployed length of flow diverting stents (FDs) to select the optimal device size remains a challenging, yet crucial, task in aneurysm treatment. This study reports on the accuracy of PreSize Neurovascular (Oxford Heartbeat Ltd), a visualization and simulation software for FD intervention planning, in predicting FD deployed length, and on its impact on device size selection.

**Methods:**

Imaging data from consecutive patients treated with Derivo Embolization Device (Acandis GmbH) were collected from University Medical Center Hamburg-Eppendorf and retrospectively analyzed. Accuracy evaluation: prediction accuracy was calculated by comparing deployed FD lengths measured from imaging data and simulated by PreSize. Size selection comparison: two Interventional Neuroradiologists (INR1, INR2), blinded to the devices deployed, used PreSize to select the optimal device size (diameter and length). Their choices were compared against the deployed devices selected by conventional planning.

**Results:**

Among 98 implanted devices, PreSize predicted deployed FD length with a mean accuracy of 94.54% (95% confidence interval [93.72%, 95.35%]). Among 98 aneurysm cases, PreSize-informed device lengths were significantly shorter (Wilcoxon signed-rank test, INR1: W = 394, P < .001, INR2: W = 305, P < .001) by 4.13 and 4.18 mm on average, and up to 20 and 25 mm, for INR1 and INR2, respectively, than the conventionally selected FDs. In 32% of cases, PreSize-informed devices resulted in fewer vessel bends covered by the FD while achieving sufficient aneurysm coverage.

**Conclusions:**

PreSize retrospectively predicted deployed FD lengths with high accuracy. Moreover, INRs in this study were more inclined to select shorter stent length in the simulation than they would have done conventionally.

## Introduction

The use of flow diverting stents (FDs) has been increasingly important in the treatment of complex wide-necked or fusiform intracranial aneurysm^1^ and FDs are being avidly developed.^2^ However, accurate sizing and prediction of proximal landing zones remain a challenging, yet crucial, preprocedural clinical task.

To select the optimal FD size for a patient, a physician needs to estimate the FD's ultimate length when deployed in that patient's anatomy. Due to the FD's braided construction, its deployed length varies widely from its nominal —also known as labeled— length, depending on the targeted vessel segment's dimension, with no demonstrable correlation between the nominal and deployed length.^3^

Selecting a device diameter that would result in sufficient wall apposition is vital for a favorable clinical outcome, yet particularly challenging due to naturally tapering vessels and vessel diameter irregularities. Undersizing can result in the device's poor vessel wall alignment, potentially leading to complications, for example, device migration or endoleaks.^4^ Conversely, excessive oversizing is undesirable and can substantially reduce the device's flow diverting abilities due to higher porosity.^5,6^

Insufficiently long FDs may show stenotic deformations at the device's proximal end, increasing the risk of thrombosis and stent stenosis.^7,8^ To avoid such outcomes and considering the challenge in accurately evaluating a specific device's deployed configuration in a specific anatomy, longer devices are often preferred to ensure that the proximal landing zone is on a straight vessel segment. However, every vessel bend the Interventional Neuroradiologist (INR) takes during FD deployment might increase the risk of stent opening failure (e.g. device twisting), which in turn may require additional deployment maneuvers, resulting in prolonged interventional durations and possibly increased ischemic event rates.^9,10^ Longer stents possibly mean covering side-branches or perforators, potentially increasing thrombotic risk and related ischemic complications.^11–14^

Traditional FD sizing is conducted based on manual measurements in two-dimensional (2D) digital subtraction angiography (DSA) and three-dimensional (3D) rotational angiography (3DRA) acquisitions of the target artery. Considering the complex device selection criteria requiring precise evaluations, current FD sizing is nonstandardized and operator-dependent.

Tools virtually simulating FD deployments for preprocedural rehearsals could assist physicians in this complex decision making. Such tools have been developed, though largely for research purposes^15,16^ or with limited in-practice use currently. Preliminary small-scale studies of such commercially available tools have indicated good prediction accuracy of deployed FD length.^3,17^ However, for virtual tools to be ultimately used consistently in clinical practice, evidence with sufficient sample sizes is required to establish the accuracy of simulation predictions and the tool's usefulness toward improving and standardizing clinical decision making.

PreSize Neurovascular (Oxford Heartbeat, UK) is a novel visualization and simulation software tool for planning FD interventions in aneurysm treatment. It provides INRs with advanced planning support, allowing them to preprocedurally “test” different devices in the patient's anatomy.

The present study aims to (i) evaluate PreSize's accuracy in predicting FD deployed length for the Derivo Embolization Devices (DED; Acandis GmbH, Pforzheim, Germany) using real patient anatomies retrospectively (henceforth referred to as *accuracy evaluation*) and (ii) assess the impact of PreSize use on selecting FD size compared to the traditional sizing approach (henceforth referred to as *size selection comparison*).

## Methods

### Study inclusion criteria

Consecutive cases of intracranial aneurysm patients (saccular or fusiform) previously treated at the University Medical Center Hamburg-Eppendorf with at least one DED between November 2015 and June 2021 were considered for this study.

Study inclusion required the availability of a minimal dataset for each intervention consisting of: (i) information of the implanted FD(s) (e.g. DERIVO-350-15 meaning a DED of 3.5 mm in nominal diameter and 15 mm in nominal length), (ii) pretreatment 3DRA of the aneurysm and adjacent vessels and (iii) post-treatment 2D-DSA images or 3DRA (or VasoCT, Philips Healthcare, the Netherlands) showing the fully opened FD(s) post-deployment.

Additionally, only cases in which the stent's proximal and distal ends were unambiguously visible were included in the *accuracy evaluation study*, to allow for the accurate calculation of the observed post-treatment deployed length. Conversely, only cases treated with one FD (no telescoping needed) were included in the *size selection comparison study*, since PreSize does not simulate telescoping.

Baseline characteristics (sex and age) and information about the aneurysm characteristics and procedure were aggregated by treating physicians.

Approval from the local Institutional Review Board (Ethik-Kommission der Ärztekammer Hamburg) was obtained and the requirement for informed patient consent was waived due to the study's retrospective nature and use of anonymized imaging data.

### Flow diverter procedure

All patients underwent pre-procedural and post-procedural DSA and 3DRA with intra-arterial injection of contrast medium (Imeron 300, Bracco Imaging Germany GmbH, Germany) using a flat panel biplane C-arm system (Allura 20/20 Philips GmbH, the Netherlands). Isotropic 3D datasets were calculated from each patient's 3D dataset. The 3DRA voxel sizes ranged between 0.11 and 0.41 mm.

Prior to intervention, all patients received double antiplatelet therapy. The procedures were performed via a transfemoral access under general anesthesia. The standard approach introduced a 7/8F guiding catheter through a short femoral sheath into the internal carotid artery or a 6/7F guiding catheter into the vertebral artery. In the anterior circulation, a triaxial approach was attempted using an intermediate catheter (Catalyst 5F, Stryker Neuroendovascular, USA). The DED was delivered through a standard 0.027” microcatheter (Headway 27, MicroVention, USA) in all cases. Vessel wall apposition was evaluated with 2D-DSA images and in some cases with a post-deployment arterial VasoCT with 50% diluted contrast medium.

### Accuracy evaluation

Cases meeting the inclusion criteria were retrospectively analyzed to assess the accuracy of PreSize deployed length predictions by comparing the PreSize-simulated FD deployed length (L_sim_) to the actual length (L_meas_) measured from the post-treatment images. The approach is detailed in Patankar et al.^18^

The following metrics were calculated for all cases included in *accuracy evaluation*:

- Relative length change between labeled FD length (L_label_) and L_meas_:
$$L_{change}=((L_{meas}-\;L_{label})/L_{label}\;)\cdot 100\text{\%}.$$
- Prediction accuracy:
$$Accuracy=(1-|L_{sim}-\;L_{meas}|/L_{meas})\cdot 100\text{\%}=100\text{\%}-Error.$$

The study's clinical investigators hypothesized that the accuracy of PreSize-simulated FD deployed length predictions would be more than 90%, as an acceptable threshold required for clinical use of such a tool.

### Size selection comparison

Two experienced INRs (5 and 10 years of INR experience referred to as INR1 and INR2, respectively), blinded to the FD size deployed in the cases included in the *size selection comparison* dataset, operated PreSize to select the optimal device size (detailed under “PreSize-informed sizing” below). The simulation-informed FD size (i.e. labeled diameter and length) was recorded and compared to the size of the FDs deployed in the actual treatment, as informed by conventional planning (detailed under “Conventional sizing method” below). Device selection in the actual procedures was performed by INRs trained and practicing in the same center and with comparable experience to INR1 and INR2.

The number of vessel bends —defined as vessel segments with bending angle lower than 135 degrees— covered by the virtually deployed FDs was also recorded for each case and compared to the number of bends covered by the implanted FDs. Finally, whether the device size automatically selected by PreSize was the INRs’ optimal size choice was recorded.

This study's clinical investigators hypothesized that the devices selected using PreSize software will be shorter than those informed by conventional planning, meeting the clinical criteria of sufficient aneurysm coverage while ensuring adequate length of proximal and distal landing zones.

### Conventional sizing method

The 3D rotational data was automatically transferred to the dedicated Philips 3D workstation. Quality of the automated 3D reconstuction was controlled by the INR. After manual delineation of the desired distal and proximal landing zone, the parent vessel in-between centerline and the approximated vessel diameter was measured using the automated stent deployment tool. Additionally, the parent vessel diameter at the distal and proximal landing zone was measured using maximum intensity projection (MIP) images reconstructed from 3DRA. The FD diameter was chosen according to the larger proximal vessel diameter. The FD length was estimated according to the centerline length, accounting for tapering of the parent vessel between the landing zones.

### PreSize-informed sizing

PreSize is a standalone software for pre-interventional planning of intracranial FD procedures. The processing steps are designed to be largely automated with minimal user input, in summary providing:

- automatic reconstruction of the 3D vasculature from 3DRA and vessel centerline visualization;
- automatic calculation and visualization of a “best-fit” deployed FD based on proximal/distal points selected by the operator along the centerline;
- manual operator adjustment of FD size and/or position for virtual rehearsal of alternative FD scenarios.

**Figure 1.**
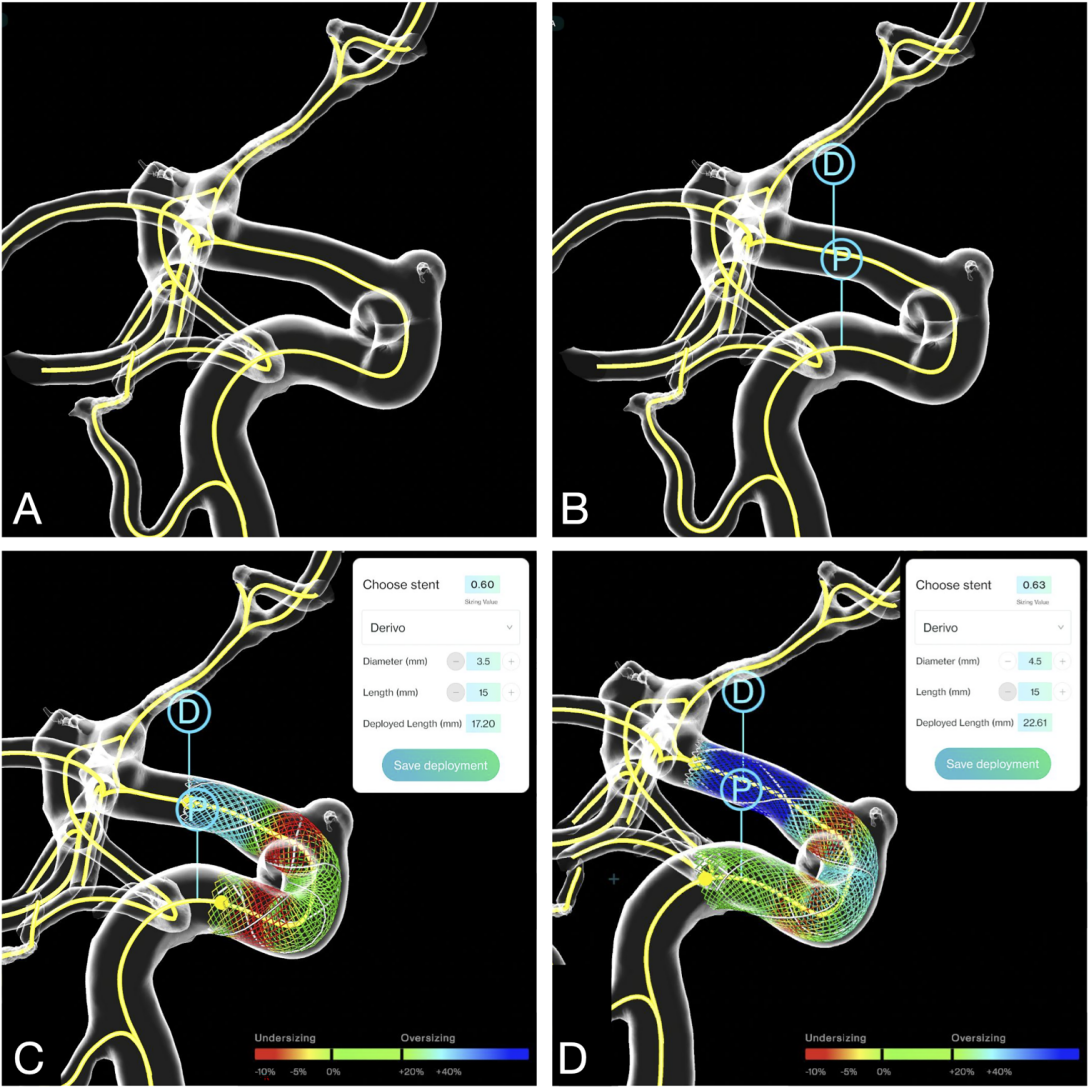
Illustrative example of using PreSize to inform flow diverter size selection. Step A: PreSize automatically reconstructs a 3D model from 3DRA images and calculates and visualizes the vessel centerline; step B: the INR selects the distal (D) and proximal (P) points along the centerline to indicate the preferred landing zone; step C: PreSize automatically calculates and visualizes a “best-fit” device size of 3.5 × 15 according to the landing zone selected at step B; step D (optional): the INR can manually make adjustments, for example increase the diameter of the simulated and visualized device (in this case to a 4.5 × 15) and move that device distally. If the INR's optimal device size is the one simulated at step C, that is PreSize's automatic best-fit selection (and counted as such in the size selection comparison study). If the INR's optimal device size is the one simulated at step D, that is a device size decision informed by PreSize (but not PreSize's automatic best-fit selection). 3D: three-dimensional; 3DRA: 3D rotational angiography; INR: Interventional Neuroradiologist.

Figure 1 illustrates processing steps (i)–(iii) and what constitutes PreSize's automatic recommendation for the *size selection comparison study* analysis. A more in-depth overview of PreSize's processing steps can be found in Patankar et al.^18^

### Statistical analysis

Equivalence testing was used for the *accuracy evaluation* to compare the *simulated* against *measured* FD deployed lengths using the two one-sided t-tests procedure (TOST) with a margin of error of 0.82 mm. This value, corresponding to twice the maximum 3DRA voxel size, was chosen as an estimate of the uncertainty introduced by the operator when placing the distal/proximal markers due to image resolution.

The Wilcoxon signed-rank test was used to assess difference in length and diameter between the optimal FDs selected with conventional planning and PreSize for the *size selection comparison*, as well as between the selections of the two INRs.

A significance level of 0.01 was chosen for the statistical analysis.

## Results

### Sample size and cohort characteristic

Eighty-eight patients (28 males, 60 females, average age = 55.2 years, age range = 14–81 years) were included in the *accuracy evaluation*, with 98 implanted DEDs analyzed (8 patients were treated with 2 DEDs, either as part of the same intervention or over different procedures, and 1 patient was treated with 3 DEDs implanted in 2 different interventions). Ten out of the 98 treated aneurysms were fusiform (10.2%) while 88 (89.9%) saccular, of which 29 were classified as wide-necked aneurysms (29.6%).

Ninety-three patients (34 males, 59 females, average age = 54.8 years, age range = 14–81 years) were included in the *size selection comparison*, corresponding to a total of 98 assessed aneurysms since 5 patients presented with multiple aneurysms. Fifteen out of the 98 assessed aneurysms were fusiform (15.3%) while 83 (84.7%) saccular, of which 31 were classified as wide-necked aneurysms (31.6%).

### Accuracy evaluation

The labeled diameter of the investigated FDs ranged between 3.5 and 5.5 mm (mean ± standard deviation = 4.36 ± 0.58 mm, median = 4.5 mm, interquartile range = 4.0–4.5 mm), and the labeled length ranged between 15 and 50 mm (mean ± standard deviation = 25.97 ± 8.24 mm, median = 25.0 mm, interquartile range = 20.0–30.0 mm).

#### Labeled length versus deployed FD length

The deployed FD length was in most cases longer than labeled, with a mean length change of 24.9% and up to 62.1%. However, a shorter deployed length up to −7.2% was observed in 7.1% of the cases.

The standard error of the linear regression between L_meas_ and L_label_ was 4.61 mm, with a 95% prediction interval of [−8.99,8.99 mm].

#### Measured versus simulated FD length

TOST equivalence testing between the measured (32.26 ± 10.76 mm) and simulated (32.40 ± 9.88 mm) deployed lengths demonstrated the equivalence between the two sets within the chosen margin of equivalence *δ* = [−0.82,0.82] mm with a significance level of 1% (t_1_(97) = 3.98, t_2_(97) = −2.80, P_1_ < .001 and P_2 _= .003).

**Figure 2.**
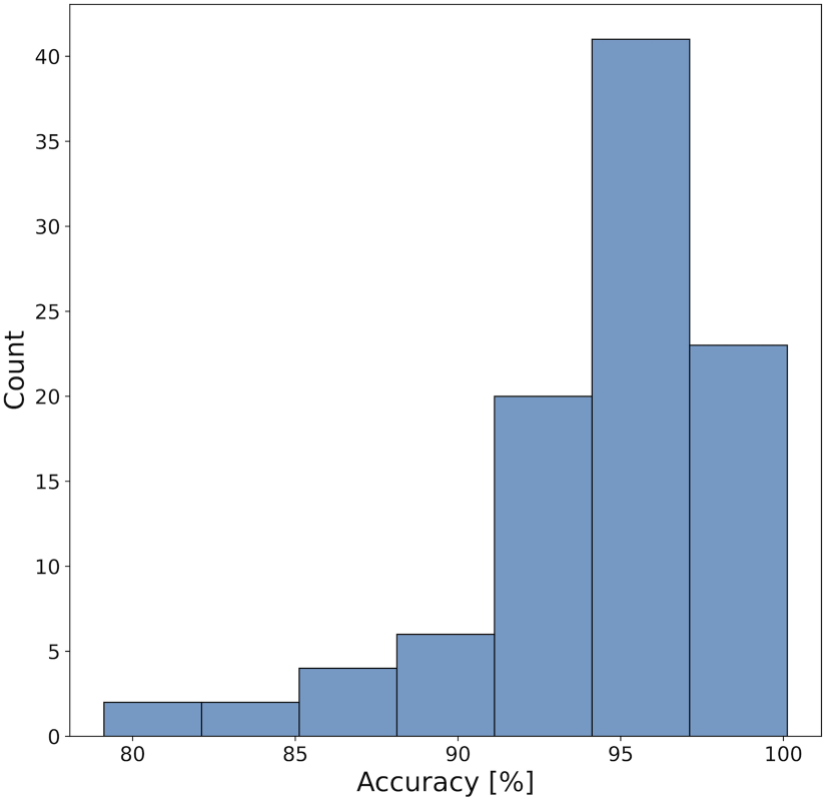
Distribution of accuracies in the prediction of DED deployed lengths obtained with PreSize. DED: Derivo Embolization Devices.

A mean accuracy of 94.54% (standard deviation of ±4.12% and median 95.50%), with 95% CI of [93.72%, 95.35%], was obtained. In 87 out of 98 processed DED deployments (88.8%), accuracy was above 90% and in 55 out of 98 (56.1%) above 95%. Figure 2 illustrates the deployment accuracy distribution.

### Size selection comparison

Nominal FD dimensions selected by conventional versus simulation-based planning (for each INR separately and for both combined) are summarized in Table 1.

**Table 1.** Dimensions of the DEDs chosen via conventional sizing and with the aid of PreSize.

	**Conventional sizing**	**PreSize-based sizing (INR1)**	**PreSize-based sizing (INR2)**	**PreSize-based sizing (INR1 + INR2)**
**FD diameter [mm]**				
Range	3.5–5.5	3.5–6.0	3.5–6.0	3.5–6.0
Mean (SD)	4.32 (0.57)	4.28 (0.70)	4.30 (0.69)	4.29 (0.70)
Median (IQR)	4.0 (4–4.5)	4.0 (3.5–4.5)	4.0 (4–4.5)	4.0 (4–4.5)
**FD length [mm]**				
Range	15–50	15–50	15–50	15–50
Mean (SD)	25.92 (8.38)	21.79 (9.17)	21.73 (9.03)	21.76 (9.08)
Median (IQR)	25 (20–30)	20 (15–26)	20 (15–25)	20 (15–25)

DED: Derivo Embolization Devices; FD: Flow diverting stent; INR: Interventional Neuroradiologist; IQR: interquartile range; SD: standard deviation.

FD size (labeled length and/or diameter) selected with PreSize was different than those selected by conventional planning in 89/98 of the investigated cases (90.8%) for INR1 and 84/98 of the cases (85.7%) for INR2; on average across the 2, in 88.3% of the cases. In all simulated cases and based on key clinical considerations for device sizing, INRs were satisfied with the aneurysm coverage and landing zone of the PreSize-selected devices. Devices selected with PreSize had a median length of 20 mm for both operators which was smaller than the median length of the DEDs chosen by conventional planning (25 mm).

PreSize-informed FDs selected by INR1 and INR2 were, on average, 4.13 and 4.18 mm, and up to 20 and 25 mm, shorter than the conventionally chosen FDs, respectively. For both operators, the FD length difference between PreSize and conventional planning reached statistical significance (Wilcoxon signed-rank test, W = 394, P < .001 for INR1, and W = 305, P < .001 for INR2).

Conversely, the median FD diameter selected was the same between the two planning approaches (4.0 mm), with no significant difference between PreSize and conventional planning separately for each operator or across the two (P = .52 for INR1, and P = .81 for INR2). Wilcoxon signed-rank tests showed no significant difference between the optimal PreSize-informed diameters (P = .25) and lengths (P = .87) selected by the two INRs.

Shorter optimal PreSize-selected devices resulted in FDs covering fewer vessel bends in 32% of the cases for both INRs. Table 2 reports a summary of the comparison between the PreSize-based and conventional sizing in terms of vessel bends covered by the deployed DED.

**Table 2.** Comparison between the number of vessel bends covered by the deployed Derivo Embolization Devices selected with PreSize and by conventional sizing.

	**INR1 selection**	**INR2 selection**
Cases with +1 covered bends compared to conventional sizing	3 (3.1%)	4 (4.1%)
Cases with same number of covered bends compared to conventional sizing	63 (64.3%)	64 (65.3%)
Cases with −1 covered bends compared to conventional sizing	30 (30.6%)	29 (29.6%)
Cases with −2 covered bends covered compared to conventional sizing	2 (2.0%)	1 (1.0%)

INR: Interventional Neuroradiologist.

INR1 and INR2 accepted the “best-fit” FD automatically suggested by PreSize based on the initial distal/proximal points selection as their optimal choice in 78% and 65% of the times, respectively (71% on average). In the remaining cases, INRs simulated alternative deployed device sizes until reaching their optimal selection.

## Discussion

### Simulation accuracy and stent elongation

This work is, to our knowledge, the first large-scale DED-specific study to evaluate prediction accuracy of a largely automated simulation software tool. The study showed higher agreement between observed and virtual deployment of the DED with PreSize compared to other studies with similar software solutions by Kellermann et al.^17^ and Narata et al.^3^ on DEDs (though both had smaller sample sizes), and by Tong et al.^19^ (though on Pipeline Embolization Devices, Covidien, Irvine, California, USA). PreSize prediction accuracy aligns well with an equivalent PreSize study on Pipeline Embolization Devices,^18^ further demonstrating PreSize's reliability.

PreSize was designed to support prospective pre-procedural decision making toward standardization of FD procedures that would minimize nonstandard deployment techniques (e.g. extreme push/pull maneuvers, ballooning), often implemented due to suboptimal device selection. Considering that such nonstandard deployment techniques are hard to accurately quantify in a virtual environment or validate, PreSize simulations represent the standard deployment. Nonetheless, PreSize deployed length accuracy has been shown to be high (i.e. 94.54% on average), and the equivalence between measured and deployed DED lengths within the margin of ±0.82 mm has been demonstrated in this study. Four out of seven outlier cases —defined as cases with accuracy lower than 87.80% (i.e. first quartile–1.5 interquartile range)— were wide-necked or large aneurysms in which intra-operative forces can lead to wider deployment configuration variability (e.g. FDs can be more or less packed or stretched in these cases).^18^ The fact that consecutive cases with aneurysms of various types (e.g. both saccular and fusiform) and sizes were examined indicates that the software's accuracy is high across a wide range of cases, representative of real clinical practice.

Separately, the large prediction interval comparing FD labeled dimensions and their deployment elongation indicates that there is no demonstrable correlation between the two, further demonstrating that the use of labeled length to predict the implanted length with satisfactory precision is limited. DED's observed elongation reached 62.1%, with a few devices (7.1%) shortened upon deployment, as similarly reported by Narata et al.^3^ Interestingly, in the equivalent Pipeline Embolization Device PreSize investigation, elongation in some cases exceeded 100%, while no devices shortened.^18^ Difference in the available device sizes, manufacturer's sizing guides and/or labeling conventions (e.g. the definition of labeled vis-à-vis stress-free diameter) prefered expression of small numbers (see comment above) devices might contribute to such difference, which further contributes to practice variability and potential challenges in preprocedural planning, especially where different device types are used. This difference also underpins the need for the separate investigation of device makes/models to evaluate differences in mechanical characteristics, behavior and labeling conventions among different devices, as well as their impact on deployment.

### Device selection

Results from the *size selection comparison* indicate that PreSize's use could lead to significantly shorter selected DEDs. This outcome may relate to increased INR confidence to choose the shortest possible FDs while maintaining sufficient coverage of the aneurysm segment when using planning tools.^20,21^ Further, this study's results suggest that shorter optimal device selection could be beneficial since it could lead to coverage of fewer bends, thus to less technically complex and faster interventions.

Previous study^20^ reported INRs’ propensity to selecting shorter devices, by 1.09 mm on average, using sizing software for Pipeline Embolization Devices. Our DED-focused study not only reproduces this finding, but reports a much more pronounced result: selection of 4.16 mm shorter devices on average, up to 25 mm shorter than conventionally chosen FDs. The difference in manufacturers’ sizing guides and available size steps between the two devices might explain this difference.

PreSize provides a simulation tool for the virtual rehearsal of different deployment scenarios for a specific case before the procedure, which could prevent the selection of longer devices than needed thus avoiding proximal landing zones across branching arteries and perforators, where feasible.^11,12^

Such tool could be helpful for new and less experienced INRs, as well as for the clinical use of new/updated FDs or the concurrent use of different FD makes/models, especially since differences among manufacturers can make sizing difficult, even for more experienced INRs.

**Figure 3.**
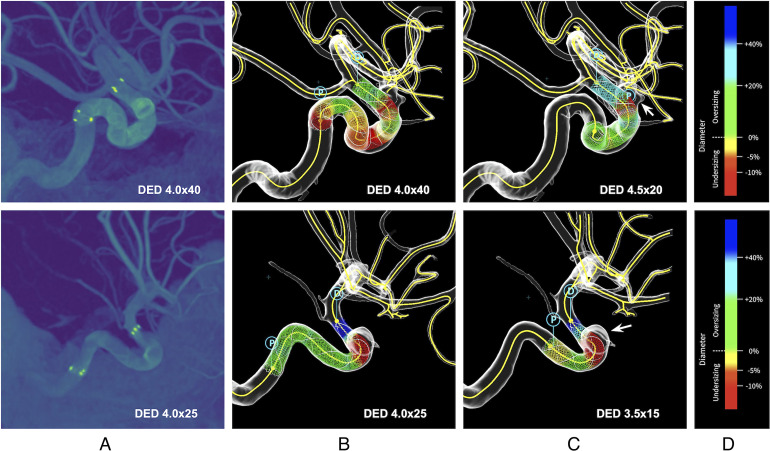
Clinical images and simulation results of two cases (first row: Case 1, second row: Case 2). (A) Maximum intensity projection of a pre-operative 3DRA co-registered with a post-operative VasoCT showing the deployed FDs (Case 1: DED 4.0 × 40, Case 2: DED 4.0 × 25). (B) Simulation results of the same FDs deployed within PreSize. (C) Virtual deployment of the optimal device chosen with the aid of PreSize by INR1 (Case 1: DED 4.5 × 20, Case 2: DED 3.5 × 15). The shorter length of the simulation-based optimal devices led to a lower number of covered vessel bends. (D) Color-bar of the FD coloring in PreSize, indicating the oversizing of the stent diameter relative to the diameter of the blood vessel. 3DRA: three-dimensional rotational angiography; DED: Derivo Embolization Devices; FD: Flow diverting stent; INR: Interventional Neuroradiologist.

Figure 3 reports two illustrative cases. In both cases, the actual deployments (Figure 3A) are closely simulated by PreSize (Figure 3B), with a deployed length accuracy of 95.7% and 98.7% for the first (top row) and second (bottom row) case, respectively. In addition, when the INRs (blinded to the actual device choices) selected the optimal DED size using PreSize, they opted for shorter devices than those that were used in practice; in both cases, they were inclined to avoid deploying past a “sharp bend” located proximally, with the PreSize-informed shorter devices showing satisfactory aneurysm coverage and landing zone (Figure 3C).

The *size selection comparison* sample included a full range of available nominal DED diameters, even scarcely clinically used large-diameter FDs (>5 mm).^22^ No statistical significance was noted in the selected FD diameter between traditional and PreSize planning, indicating that PreSize's segmentation algorithms closely resemble diameter evaluations of experienced INRs.

Interestingly, when examining the approaches using PreSize by the two INRs, it was noted that one INR was more frequently inclined to accept the PreSize automatically calculated “best-fit” device based on the selected proximal/distal landing points, as opposed to manually modify the device size. At first glance, this observation might indicate practice variability. However, there is no statistically significant difference between the devices ultimately selected by the two INRs, in fact indicating practice standardization. Though physicians might operate PreSize in slightly different ways (e.g. selecting the landing zone in a slightly different way), PreSize assists toward consistent devices choices according to generally accepted clinical criteria for optimal stent deployments.

### Limitations

The focus of this retrospective work was a validating step to establish the reliability of simulations on a wide range of cases representative of real clinical practice, as well as to estimate the impact of such a dedicated software on planning outcome, in particular, device selection. But further prospective research is required to confirm clinical impact. A UK-wide prospective PreSize study is currently under way to investigate the impact of PreSize in real clinical settings (trial registration number ISRCTN17947655).

Given other braided stents do not have the same behaviors to DED, this study's findings cannot be directly extrapolated to other manufacturers’ FDs. Nevertheless, this study focused on the DED flow diverter which allowed for the first-of-its-kind large-scale study for this device. Particularly, the size selection evaluation indicates potential value for the use of the software in braided stent selection more generally.

## Conclusions

PreSize virtual simulation software has potential to assist INRs during FD sizing with high accuracy. In this retrospective study INRs were more inclined to select shorter stent length in the software simulation and visualization than they would have done conventionally. For some instances, shorter devices resulted in fewer vessel bends covered by FDs. The potential benefits of using the shortest devices necessary for sufficient aneurysm coverage and safe stent deployment suggest that virtual FD deployment simulations to drive device selection could be valuable in the workflow for all FD procedures and could lead to more consistent and standardized, albeit patient-specific, clinical practice.

## Abbreviation list

2D-DSA: Two-dimensional digital subtraction angiography
3DRA: 3D rotational angiography
FD: Flow diverting stent
DED: Derivo Embolization Device
INR: Interventional Neuroradiologist
MIP: Maximum intensity projection
